# Bladder management methods and urological complications in spinal cord injury patients

**DOI:** 10.4103/0019-5413.77134

**Published:** 2011

**Authors:** Roop Singh, Rajesh Kumar Rohilla, Kapil Sangwan, Ramchander Siwach, Narender Kumar Magu, Sukhbir Singh Sangwan

**Affiliations:** Department of Orthopedic Surgery, Paraplegia and Rehabilitation, Pt. B.D. Sharma PGIMS, Rohtak, Haryana, India

**Keywords:** Spinal cord injury, urinary bladder, clean intermittent catheterization, urological complications, indwelling catheterization

## Abstract

**Background:**

The optimal bladder management method should preserve renal function and minimize the risk of urinary tract complications. The present study is conducted to assess the overall incidence of urinary tract infections (UTI) and other urological complications in spinal cord injury patients (SCI), and to compare the incidence of these complications with different bladder management subgroups.

**Materials and Methods:**

545 patients (386 males and 159 females) of traumatic spinal cord injury with the mean age of 35.4±16.2 years (range, 18 – 73 years) were included in the study. The data regarding demography, bladder type, method of bladder management, and urological complications, were recorded. Bladder management methods included indwelling catheterization in 224 cases, clean intermittent catheterization (CIC) in 180 cases, condom drainage in 45 cases, suprapubic cystostomy in 24 cases, reflex voiding in 32 cases, and normal voiding in 40 cases. We assessed the incidence of UTI and bacteriuria as the number of episodes per hundred person-days, and other urological complications as percentages.

**Results:**

The overall incidence of bacteriuria was 1.70 / hundred person-days. The overall incidenceof urinary tract infection was 0.64 / hundered person-days. The incidence of UTI per 100 person-days was 2.68 for indwelling catheterization, 0.34 for CIC, 0.34 for condom drainage, 0.56 for suprapubic cystostomy, 0.34 for reflex voiding, and 0.32 for normal voiding. Other urological complications recorded were urethral stricture (n=66, 12.1%), urethritis (n=78, 14.3%), periurethral abscess (n=45, 8.2%), epididymorchitis (n=44, 8.07%), urethral false passage (n=22, 4.03%), urethral fistula (n=11, 2%), lithiasis (n=23, 4.2%), hematuria (n=44, 8.07%), stress incontinence (n=60, 11%), and pyelonephritis (n=6, 1.1%). Clean intermittent catheterization was associated with lower incidence of urological complications, in comparison to indwelling catheterization.

**Conclusions:**

Urinary tract complications largely appeared to be confined to the lower urinary tract. The incidence of UTI and other urological complications is lower in patients on CIC in comparison to the patients on indwelling catheterizations. Encouraging CIC; early recognition and treatment of the UTI and urological complications; and a regular follow up is necessary to reduce the medical morbidity.

## INTRODUCTION

The increased survival of SCI patients via the modern spinal unit care is associated with secondary complications, which continue to pose management challenges. Urinary tract infection (UTI) is still one of the leading cause of morbidity in SCI patients.^1^–^6^ Urinary and skin complications are the two main reasons for hospital readmissions in people with chronic SCI.^7^ UTI interferes with the rehabilitation, and may lead to secondary urologic complications.^2^ Recurrent urinary tract infections, indwelling catheters, vesicoureteral reflux, and immobilization hypercalcuria are a few of the major risk factors for the development of urolithiasis among spinal cord injury patients.^8^ Waites *et al*. reported that the incidence of significant bacteriuria, defined as more than 10^5^ colony-forming units per milliliter of urine, was 18.4 episodes per person-year, while the rate associated with fever was 1.82 episodes per person-year.^9^ Siroky reported that the overall rate of urinary infection in a SCI patient was about 2.5 episodes per patient, per year.^4^ Several factors appear to be responsible for an increased risk of infection in the neurogenic bladder.^2^,^4^ Altered voiding dynamics, elevated intravesical pressure, and use of urinary drainage catheters contribute to an increased risk of symptomatic urinary tract infection.^2^–^4^ Frequent exposure to antibiotics increases the risk of infection by resistant organisms.^2^,^3^

Many different bladder management methods are now in use in SCI patients; the most common among them are intermittent catheterization by a trained staff or self, suprapubic cystostomy, indwelling catheterization, condom drainage, Crede maneuver, and ileal conduit. Each of these methods has certain advantages and disadvantages in terms of convenience, expense, and differential risk of a variety of secondary complications such as urinary tract infection, orchitis, epididymitis, penoscrotal abcess and fistula, penile pressure ulcers, and bladder and renal calculi.^10^ The optimal bladder management method should preserve renal function, minimize urinary tract complications and the risk of urothelial neoplasm. In addition, patient comfort, convenience, and quality of life are important factors in bladder management decisions.^11^ The present study aims to assess the overall incidence of UTI and other urological complications in SCI patients; and to compare the incidence of these complications in different bladder management subgroups.

## MATERIALS AND METHODS

The present study was conducted prospectively between 1995 and 2007, in patients with spinal cord injury(both acute and chronic), presenting to a tertiary level referral center. All patients 18 years or more with post traumatic SCI and injury below C4 who had neurological deficit along with bladder involvement at the time of injury and could be followed up for a minimum of one year were included in this study. The chronic SCI patients with bladder involvement who were followed prospectively for a minimum one year after enrolment into the study were included in the analysis.

The protocol followed for patients with acute SCI includes indwelling catheterization at the time of admission. Clean intermittent catheterization (CIC) is then taught to the patient and his / her attendant, usually after two to four weeks. The patients who accept this method are kept on this mode of bladder management permanently or till they develop normal voiding. Patients not accepting this mode of bladder management are kept on indwelling catheters or advised / kept on other alternative methods like condom drainage, suprapubic cystostomy, and reflex voiding (Spontaneous / Crede maneuver). Urine cultures and leukocyte counts in the urine are routinely performed every 15 days during the time of hospitalization, when the patient shows clinical signs of UTI, as also on the subsequent follow-ups. The tip of the indwelling catheter (if it needs to be replaced at the time of culture) is also sent for microbiological examination. Leukocyte counts are conducted in the sediment of the centrifuged urine and the results are expressed as leukocytes per high power field. Other methods prevalent during the study period, included in the protocol, were intermittent clamping of catheter, bladder irrigation, change of pH, and plenty of fluids orally. After discharge from the hospital, the patients were usually followed-up in the Outpatient Department twice during the first six months, at one year, and six monthly until the last follow-up. The patients were instructed to come for follow-up and admission on development of signs of UTI or any other complication or any adverse event related to the urinary drainage system. Routine check-up investigations included complete urine analysis, ultrasonography of the abdomen, and urine cultures.

The protocol for patients with chronic SCI depended on the bladder management method the patient was following. At first attendance patients who did not recover from bladder involvement were encouraged to use CIC. Patients not accepting CIC were left with the bladder management method that they are following. Urine cultures and leukocyte counts in the urine were routinely performed at first attendance, when the patient shows clinical signs of UTI, as also on the subsequent follow-ups. They were followed twice next six months, at one year, and six monthly until the last follow-up. Rest of the protocol was same as for patients with acute SCI.

A total of 545 patients (386 males and 159 females) met the inclusion criteria. The mean age of the patients was 35.4±16.2 years (range, 18 – 73 years). A total of 382 patients with acute SCI presented within a few hours to the first three months of the traumatic event. Patients with chronic SCI presented at a mean of 20.6±9.2 months (range: 3 months to 5 years) after injury. The patients were given detailed information about the purpose of the study and written consent was obtained from all the participants. To begin with, a complete history of all the patients was recorded, followed by a complete general physical and neurological examination. The grade of neurological deficit in SCI was classified according to the American Spinal Injury Association Impairment Scale (AIS).^12^ Neurological deficit as per AIS at the time of inclusion into the study and level of injury are shown in Table 1. There were 381 complete (AIS A) and 164 incomplete (AIS B, C, and D) neurological injuries. Average follow-up was 16.4 months (range, 12 – 25 months).

**Table 1:** Level of injury and neurological deficit

Neurological level	Complete (AIS A)	Incomplete (AIS B, C, and D)	Total no. pts
C4–C8	121	64	185
D1–D10	81	12	93
D11–L2	116	86	202
Below L2	31	34	65
Total	349	196	545

AIS: American Spinal Injury Association Impairment Scale^12^

The bladder management method was assigned as a method predominantly used, based on the history and records of the treatment received for the majority from time since injury in chronic SCI, and during the first year in acute SCI patients. In 40 patients of SCI, the bladder had recovered and they were voiding normally. Standard laboratory methods were used for quantitative urine culture and the isolation and identification of organisms. The patients were asked to keep a record of the complications if they failed to attend our institute for the complication. Patients having urological complaints were further appropriately investigated for hematological analysis, culture studies, radiographs, ultrasonography, and cystoscopy. The Surgery and Urology Departments were also consulted to diagnose and treat urological complications like urethral stricture, fistulae, and abscesses.

Urinary tract infection was defined as a colony count of 10 ^5^colony forming units per milliliter or greater, with a fever of 38° and two symptoms including, over distention of the bladder, lower abdominal pain, increased urinary incontinence, increased spasticity, autonomic hyperreflexia, and increased sweating or malaise.^13^,^14^ Asymptomatic bacteriuria was defined as a colony count of 10^5^ colony forming units per milliliter or greater, without fever or other symptoms.^14^ We assessed the incidence of UTI and bacteriuria as the number of episodes per hundred person-days. A total of 100 person-days is equivalent to 100 persons followed for one day, who were free of urinary tract infection or bacteriuria throughout the day.^13^ Incidence was defined as the number of new cases of UTI or bacteriuria in a population during a day, divided by the total number of 100 person-days that the population was at risk for the condition. We assessed the incidence of other urological complications as percentages.

## RESULTS

Bladder management methods included indwelling catheterization in 224 cases, CIC (reusable catheters) in 180 cases, condom drainage in 45 cases, suprapubic cystostomy in 24 cases, and reflex voiding (Spontaneous / Crede maneuver) in 32 cases. Forty patients of SCI bladder had recovered and they were voiding normally. The mean period of indwelling catheterization from time of injury to change of bladder management was 25 days (range, 11 – 64 days) in acute SCI patients. The overall incidence of bacteriuria of all drainage methods was 1.70 / hundered person-days. The overall incidence of UTI of all drainage methods was 0.64 / hundered person-days. The incidence of bacteriuria and UTI with various drainage methods is depicted in Table 2 and the incidence of other urological complications observed in the present study are shown in Table 3. Incidences of urethral stricture (*P*=0.036), urethritis (*P*=0.036), periurethral abscess (*P*=0.03), epididymorchitis (*P*=0.04), and hematuria (*P*=0.005) were less (statistically significant) in the CIC group when compared with the indwelling catheterization group, whereas, the incidence of incontinence was more (*P*<0.001) in the CIC group when compared with the indwelling catheterization group. The condom group patients had less urethral strictures than the indwelling catheterization group (*P*=0.04), but had more incontinence (*P*<0.001) than the CIC group and normal voiding group. The Crede’s maneuver group had more incidence of lithiasis (*P*=0.002) than the CIC (*P*=0.002) and indwelling catheterization groups (*P*=0.048); and also had more incontinence (*P*=0.001) than the normal voiding group. The suprapubic cystostomy and normal voiding groups had more rates of incontinence than the indwelling catheterization group (*P*<0.001)

**Table 2:** Incidence of urinary tract infection and bacteriuria with various bladder management methods

Bladder management type	No. of patients	Incidence (100 person-days) bacteriuria	Incidence (100 person-days) UTI
Indwelling catheterization	224	4.02	2.68
Clean intermittent catheterization	180	1.02	0.34
Condom	45	2	0.34
Reflex voiding (Spontaneous / Crede maneuver)	32	1.20	0.44
Suprapubic cystostomy	24	0.80	0.56
Normal voiding	40	0.26	0.32
Overall	545	1.70	0.64

UTI: Urinary tract infections

**Table 3:** Other urological complications observed in the present study

Urological complications	Incidence (%)							
	Overall (545) number (%)	*P* value between all groups	Indwelling catheterization (224)	CIC (180)	Condom (45)	Reflex voiding (Spontaneous / Crede maneuver) (32)	Suprapubic cystostomy (24)	Normal Voiding (40)
Urethral stricture	66 (12.1)	SS (0.04)	40# (*P*=0.036)	18+ (*P*=0.036)	2+ (*P*=0.04)	2	1	2
Periurethral abscess	45 (8.2)	NS	26# (*P*=0.03)	9+ (*P*=0.03)	5	2	2	1
Epididymorchitis	44 (8.07)	NS	25# (*P*=0.04)	9+ (*P*=0.04)	4	2	2	2
Urethral false passage	22 (4.03)	NS	9	9	2	1	1	
Urethritis	78 (14.3)	NS	#43#*(*P*=0.036), *(*P*=0.048)	20+ (*P*=0.036)	8	3	2	2+ (*P*=0.048)
Urethral fistula	11 (2)	NS	7	3			1	
Lithiasis	23 (4.2)	NS	9	4	2	5#+ #(*P*=0.002) +(*P*=0.048)	2	1
Hematuria	44 (8.07)	NS	27# (*P*=0.005)	7+ (*P*=0.005)	4	2	2	2
Incontinence	60 (11)	NS	3#*#(*P*=0.001) *(*P*=0.0009)	15+ (*P*=0.001)	17#*#(*P*<0.001) *(*P*=0.016)	16* (*P*=0.001)	4+ (*P*<0.001)	5+ (*P*<0.001)
Pyelonephritis	6 (1.1)	NS	1	1	1	2	1	

CIC: Clean intermittent catheterization, SS: Statistically significant, NS: Not significant, Chi square with Yates’ correction was used for statistical analysis. P value< 0.05 was considered significant.,

# shows the statistically significant difference of the complication between this bladder management group and the CIC group,

* shows the statistically significant difference of the complication between this bladder management group and the normal voiding group, + shows the statistically significant difference of the complication between this bladder management group and the indwelling catheterization group.,

$ Figures in parentheses in this column are in percentage

We also observed discharge around the catheter in 188 (84%) and frequently blocked catheter in 69 (30.8%) cases of indwelling catheterization, and difficulty in removal of Foley’s catheter requiring suprapubic or perineal puncture of the balloon in 18 (8.03%) cases. The passage of amorphous material in the urine was observed in almost all the patients on indwelling catheterization. Stress incontinence was observed in 60 patients (11%) and a majority of these patients were females (41 patients; 68.3%)

Total positive urine cultures were 1808 (mean 3.3/ per person). Escherichia coli were the predominant isolates in 298 cultures (16.5%), followed by *Klebsiella* in 217 (12%) *Staphylococcus aureus* in 144 (8%), and *Pseudomonas aeruginosa* in 144 cultures (8%). Most of the time the organisms had more than one isolate (polymicrobial) and were resistant to commonly used antimicrobial agents. During the initial five years of the present study, the indwelling catheter was the most common mode of bladder drainage, and the acceptance of CIC was only 11% of the 174 patients; but at the end of the study acceptance had risen to 33% of all the 545 patients enrolled. The majority of urological complications (58.6%) were observed in the initial seven to eight years of study.

## DISCUSSION

Urinary tract infection (UTI) is responsible for major morbidity in SCI patients.^1^–^6^ Despite improved methods of treatment, urinary tract morbidity still ranks as the second leading cause of death in SCI patients.^15^ Spinal cord injury produces profound alterations in the lower urinary tract function.^4^ Incontinence, elevated intravesical pressure, reflux, stones, and neurological obstruction, commonly found in the spinal cord-injured population, increase the risk of UTI.^4^ Incomplete voiding and catheter use contribute to an increased risk of symptomatic UTI.^2^ Medical morbidity and urological outcome in SCI patients, using different methods of bladder management, is made difficult by the many uncontrollable management variables within each method, which may influence the outcome. It remains difficult to get a proper estimate of the risk of infection from literature. The data differ so much, that many different factors must influence the prevalence of this complication. The present study aims to assess the incidence of the UTI, and other urological complications in spinal cord injury patients on different bladder management methods so that a better bladder rehabilitation protocol can be adopted.

Incidence of UTI in patients with indwelling catheterization in the present study is 2.68/ hundered [Tables 2 and 3] person-days and is comparable to other series.^13^,^16^ Esclarin De Ruz *et al*. reported an incidence of UTI in male indwelling catheterization as 2.72/ hundered person-days,^13^ while Ericksson *et al*. observed an incidence of 2.10 / hundered person-days.^16^ Incidence of bacteriuria in patients with indwelling catheterizations in the present study is 4.02 / hundered person-days, which is comparable to 5 / hundered person-days in the studies reported in literature.^13^ The most prevalent risk indicator of UTI in SCI patients is an indwelling catheter.^5^ The risk of UTI increases with the increasing duration of catheterization.^17^ There is now an appreciation of the fact that a creeping adherent biofilm of bacteria frequently ascends through the luminal and external surfaces of an indwelling catheter, often within 8 to 24 hours, leading to bacterial adherence to the bladder surface, correlating with the symptomatic infection.^4^ On account of chronic bacterial infection within the biofilms, an antibacterial treatment based on the urinary culture of bacteria in the urine and its antimicrobial susceptibility may fail to eradicate catheter-associated UTI.^5^ In cases of recurring UTI in patients with a permanent urinary catheter, it may be beneficial to change the catheter every one or two weeks.^5^

The incidence of UTI in the present study (0.34) is comparable to 0.41 – 2.95 / hundred persondays reported in the literature.^13^,^16^ The incidence of bacteriuria in patients on CIC in the present study (1.02) is slightly lower as compared to 2.95 / hundered person-days, the incidence reported in the previous study.^13^ CIC in the rehabilitation setting does not appear to place the patient with SCI at an increased risk for developing symptomatic UTI, and has significant cost and time saving benefits for the healthcare system, and it also enhances the transition of the patient from rehabilitation to community.^17^

The incidence of UTI in patients on condom drainage is 0.34 / hundered person-days in the present study, which is comparable to 0.36 / hundered person-days reported by Esclarin De Ruz *et al*.^13^ The incidence of bacteriuria on condom drainage is 2.0 / hundered person-days in the present study, whereas, Esclarin De Ruz *et al*. reported an incidence of 2.41 / hundered person-days.^13^ This method is good; but associated with increased residual volume, and close monitoring is required.^13^ The incidence of UTI is low in the present study in patients on CIC (0.34) and condom drainage (0.34), when compared to those on Crede maneuver (0.44), suprapubic cystostomy (0.56), and indwelling catheterization (2.68). It suggests that CIC and condom drainage, are reasonable means of managing neurogenic bladder dysfunction with respect to UTI. However, patients on condom drainage had more incidence of incontinence in comparison to those on CIC. Normal voiding was associated with the least incidence of bacteriuria and UTI.

The overall incidence of UTI in the present study is 0.64 / hundered person-days, which is comparable to 0.68 / hundered person-days reported by Esclarin De Ruz *et al*.^13^ The overall rate of UTI in SCI patients is about 2.5 episodes per patient per year.^4^ The overall incidence of bacteriuria of all drainage methods was 1.70 / hundered person-days in comparison to 2.72 / hundered person-days in a study by Esclarin De Ruz *et al*.^13^ It is generally believed that due to various unavoidable circumstances such as cost constraints, non-availability of specialized care, and lack of skills and specialized training, the complication rate is high in developing countries. The reasons for a comparable rate of complications in the present study may be due to the fact that these patients were followed-up in a tertiary care center for a longer duration, in a prospective manner. Patients and their attendants / caretakers were also educated to avoid these complications and report to the hospital early, in the event of occurrence of these complications. Asymptomatic bacteriuria is very frequent in patients treated by intermittent catheterization and does not justify antibiotic therapy, as antiseptics and urinary alkalinizers or acidifiers have been shown to be effective.^1^ Antibiotic treatment should be limited to symptomatic UTI and initiated after sensitivity testing only.^18^ Empiric use of antibiotics must be limited to highly symptomatic infections until the results of sensitivity testing are available.^18^ *Escherichia coli, Pseudomonas spp*., *Klebsiella spp*., Proteus spp., Serratia spp., Providencia spp., Enterococci, and Staphylococci are the most frequently isolated bacteria in urine specimens taken from individuals with SCI.^6^,^19^ We also had a similar distribution of isolates in the urine cultures in SCI patients.

We had lowest incidence of complications in patients on CIC for neurogenic bladder. CIC has been reported as the safest bladder management method for spinal cord injured patients, in terms of urological complications in other series also.^11^ The catheterized group experienced significantly more problems with renal damage, recurrent urinary tract infection, stones, and urethral complications.^20^ Elimination of indwelling urinary catheters in patients with SCI will significantly reduce the incidence of urinary tract complications and lead to a better preservation of renal function.^20^ If the patient has a reinfection or relapsing symptomatic UTI, it is important to check for inadequately treated infection and complications, which need special attention, in particular residual urine and urinary stones.^6^ CIC in the rehabilitation setting does not appear to place the patient with SCI at an increased risk for developing symptomatic UTI, and has significant cost and time saving benefits for the healthcare system, and it also enhances the transition of the patient from rehabilitation to community.^17^

Recurrent UTI, indwelling catheters, vesicoureteral reflux, and immobilization hypercalcuria are a few of the major risk factors for the development of urolithiasis among spinal cord injury patients.^8^ In a study by Favazza *et al*., in SCI patients, when compared with the control group, the patients forming bladder stones were older (*P*=0.03), were more likely to have indwelling catheters (*P*<0.0001), had a history of infections with urease-producing organisms (*P*=0.04), and had complete injuries (*P*=0.018).^21^ Chang *et al*. reported a high prevalence of urological complications in patients using the Crede maneuver. They reported pyuria in 82.4%, urinary lithiasis in 31.3%, ureteral dilatation in 59.5%, hydronephrosis in 35.1%, and renal damage in 16.2% of the patients.^22^ However, the rates of urological complications with this method were lower in the present study. This may be partly due to the small number of patients (6% patients) using this bladder management method for a shorter period in the present study and partly due to the prospective nature of the study, as patients were instructed to take precautions to avoid urological complications. Gallien *et al*. are also of the opinion that percussion and Crede maneuver appear to be the acceptable techniques of bladder management, if the patient is closely monitored.^23^ Weld and Dmochowski observed the effect of bladder management on urological complications in 316 posttraumatic SCI patients; of the 398 complications 236 developed in 61 (53.5%) patients on chronic urethral catheterization, 57 in 25 (27.2%) on clean intermittent catheterization, 57 in 24 (32.4%) who voided spontaneously and 48 in 16 (44.4%) on suprapubic catheterization. They reported epididymitis in 16.1%, Pyelonephritis in 3.5%, upper tract stone in 35.1%, urinary bladder stones in 14.6%, periurethral abscess in 2.8%, urethral stricture in 11.7%, and vesicoureteral reflex in 15.8%. The intermittent catheterization group had statistically significant lower complication rates compared to the urethral catheterization group and no significantly higher complication rates relative to all other management methods for each type of complication studied.^11^ Incidence of periurethral abscess and urethral fistulae in the present study was more compared to this study. This might be because a large number of patients were on indwelling catheterization and patient-related factors like personal hygiene, fluid intake, and catheter care. We agree with them that inappropriate selection of the bladder management method not only adversely affects a patient’s quality of life, but also has a significant detrimental impact on the economic status of the healthcare system. Larsen *et al*. also reported more significant problems, with renal damage, recurrent urinary tract infection, stones, and urethral complications.^24^ For those patients who require long-term urinary appliances, patient education, and strict attention to hygiene and catheter-care policies, it is important to prevent urological complications.^25^ In the present study, the incidence of the urological complications reduced in the later phase of the study; it might be multifactorial. Increase in acceptance for CIC; proper hygiene and catheter care; high fluid intake; patient education; and overall change in the aptitude of the patients toward bladder management methods could be the possible reasons for this. This may also be due to the regular follow-up of the patients enrolled for this prospective study as they were given advice to prevent urological complications regularly.

The limitations of our study include a non-randomized nature. In addition, the study is limited because each patient was categorized by a predominant bladder management, defined as the method used for the majority of time since injury. Most likely the strict categorization of patients into a single predominant bladder management group may have introduced experimental error. However, the large number of patients and overall longer follow-up helped to minimize the influence of this variable.

## CONCLUSION

Urinary tract complications continue to cause significant morbidity after SCI and largely appear to be confined to the lower urinary tract in the whole series; although reflex voiders and those on condom drainage had a slightly higher incidence of upper urinary tract complications (pyelonephritis and lithiasis) compared to the persons using other methods of bladder management. The incidence of UTI and other urological complications is lower in patients on CIC when compared to patients with indwelling catheterizations. A more aggressive attitude in urinary management following SCI is necessary in order to improve the rehabilitation and quality of life of persons with SCI.
